# Factors associated with the workload of health professionals in hospital at home: a systematic review

**DOI:** 10.1186/s12913-022-08100-4

**Published:** 2022-05-26

**Authors:** José A. Cordero-Guevara, Naiara Parraza-Díez, Kalliopi Vrotsou, Mónica Machón, Estibalitz Orruño, Miren J. Onaindia-Ecenarro, Manuel Millet-Sampedro, José Regalado de los Cobos

**Affiliations:** 1 Epidemiology and Public Health Group, Bioaraba Health Research Institute, C/ Isabel Orbe s/n., 01002 Vitoria-Gasteiz, Araba/Álava, Spain; 2 REDISSEC, Health Services Research On Chronic Patients Network, Madrid, Spain; 3 Kronikgune Institute for Health Services Research, Barakaldo, Bizkaia Spain; 4 Primary Care Group, Biodonostia Health Research Institute, Donostia-San Sebastian, Spain; 5 Biocruces Bizkaia Health Research Institute, Barakaldo, Spain; 6 Osakidetza Basque Health Service, Galdakao University Hospital, Home Hospitalisation Unit, Galdakao, Spain; 7 Osakidetza Basque Health Service, Bidasoa Hospital, Home Hospitalisation Unit, Hondarribia, Spain; 8 Osakidetza Basque Health Service, Araba University Hospital, Home Hospitalisation Unit, Vitoria-Gasteiz, Spain

**Keywords:** Workload, Home Care Services, Hospital at Home, Associated Factors, Systematic Review

## Abstract

**Background:**

Understanding the factors related to workload, could help hospital at home (HaH) managers to make decisions on the most appropriate and efficient use of the HaH services. Published studies on this topic are scarce, so we have conducted a systematic review to identify such factors according to published evidence.

**Methods:**

Due to the heterogeneity of HaH models, HaH was defined as a care that provides a set of medical and nursing care and attention of hospital rank to patients at home, when they no longer require hospital infrastructure but still need active monitoring and complex care. The electronic data base literature search was conducted in MEDLINE (Ovid), EMBASE (Ovid), and Cinahl (EBSCOhost) from inception to December 2021, including grey literature. Search terms related to `hospital at home´, `workload´ and `care time´ were used. There was no restriction on language, type of study or year of publication. Quality of included studies was assessed using the Critical Appraisal Skills Programme (CASP) checklist and certainty in the body of evidence was assessed using the GRADE Pro Tool. Results were summarised in a tabulated format.

**Results:**

Eighteen studies with 56,706 patients were included. Workload was measured as time, number of visits or both. The predictive factors of the workload included variables related to patient characteristics and other valid and reliable patient classification systems, as well as characteristics of the institutions where the studies were conducted. The factors associated with higher workloads were: being older, male, living in a rural environment, presenting a higher number of diagnoses, having worse functional status and being unable to assume self-care.

**Conclusions:**

The identified predictors of workload are mostly associated with home nursing care. The results could be useful and applicable to different organisational models of HaH health systems. More studies that include physicians and proxy measures of workload are needed.

**Supplementary Information:**

The online version contains supplementary material available at 10.1186/s12913-022-08100-4.

## Background

Different health care models have been labelled as hospital at home (HaH) in the international literature over time. Such models vary according to the general organisational guidelines of the health system in each country and according to the funding and the profile of the patients (or diseases) for which care is provided [1–3]. Heterogeneity is also observed in the type of team in charge of coordinating the care (e.g., hospital vs community-based), the care team composition (e.g., nurses, physiotherapists, occupational therapists), the programme components (e.g., additional elements such as patient and caregiver education), the number of home visits, the availability of after-hours support and specific medical services provided (e.g., home oxygen, intravenous fluids) [4, 5].

In addition, scientific evidence shows a broad terminology to refer to HaH. UK Hospital at Home Society [6] and some authors have defined HaH as “ the care service offered for a limited period of time that provides active treatment by health care professionals in the patient’s home, for a condition that otherwise would require acute hospital in-patient care” [7–9]. In Spain, HaH is defined as “a care alternative that consists of an organized model capable of providing a set of medical and nursing care and attention of hospital rank (provided by health professionals and material resources of the hospital itself), both in quality and quantity to patients at home, when they no longer require hospital infrastructure but still need active monitoring and complex care [3].

There are two main types of HaH programmes—early supported discharge (ESD) and admission avoidance (AA) [5]. ESD aims to accelerate the discharge of admitted patients, thus, partially substituting hospital care. AA directly admits patients into HaH based on general practitioner referrals, thereby avoiding physical contact with the hospital, or through direct admissions from the emergency room without inpatient stay. According to the systematics reviews conducted by Shepperd et al. [8, 10], there are some benefits that could be obtained with this type of service: reductions at 6 month mortality, rate of hospital readmissions and average hospital stay; improved daily living functional outcomes and quality of life; reduced stress and additional burden on caregivers; and finally reduced costs. Nevertheless, there is a limited evidence on the effectiveness of these types of programs as an alternative to inpatient care and the cost-effectiveness is uncertain. Difficulties relating to definitions of HaH may have reduced the effect attributable to substitution [7, 9], although some benefit was observed [11]. Other authors have reviewed the effect of HaH schemes for patients with specific conditions, such as COPD and heart failure and have found that HaH may be advantageous with respect to readmission-rates in these patients [12–15].

The availability of resources in the HaH service depends on the number of users, as well as the workload that these users entail. Understanding the factors related to workload, such as the volume and type of care administered to the patients, will offer a better knowledge of the HaH operational mechanisms and will indicate areas for improvement. Thanks to this information, HaH managers could anticipate the needs, as well as support decision making regarding the weight and composition of the teams in the short or long-term. This knowledge could indeed lead to a more appropriate and efficient use of the HaH services.

There are a few studies aimed at identifying the factors that influence workload or workload intensity [16, 17] associated to home-based care and the literature on HaH in particular is scarce. So we conducted a systematic review to identify the factors associated with workload in HaH defined by the Spanish HaH society.

## Methods

A systematic literature review was conducted following the principles of the PRISMA statement [18].

The study was approved by the local Ethics Committee. A study protocol was prepared, but the review was not registered. The datasets used and/or analysed during the current study are available from the corresponding author on reasonable request.

### Patient and public involvement

The systematic review focuses on workload factors from the point of view of the organisation of the HaH health service, which does not directly involve the patient. However, despite no patient and public involvement was considered in the design, conduct, reporting or dissemination plans of our research, the results will be communicated to both, health managers and the community. Furthermore, the principal investigator of this research as well as most collaborators, are HaH doctors and nurses with many years of experience. The point of view of these healthcare professional was carefully considered during all phases of the present work.

### Search strategy

The structured research question was: “What are the factors that influence the workload of health professionals in the setting of HaH?” which was formulated in the PEO (Population, Exposition, Outcome) format to adequately frame the research question:

- P: health professionals (nurses and doctors) in charge of HaH.
- E: factors associated to the workload of health professionals.
- O: the main outcome measure was the workload or care burden of health professionals expressed as time attributed to each activity, number of visits or intensity of care measured through specific measurement instruments or patient classification systems.

Two authors conducted the electronic data base literature search using MEDLINE (Ovid), EMBASE (Ovid), and Cinahl (EBSCOhost), from inception to December 2021. The search was based on the combination of free-text keywords and indexing terms (MeSH) related to the terms `hospital at home´, `workload´ and `care time´ (Table S1). Additionally, inverse searches were carried out by revising the reference lists of the included articles in order to identify additional relevant studies that were not retrieved by the automatic searches. There was no restriction on language, type of study or year of publication.

A complementary grey literature search was also carried out, which included the following sources via internet: the Spanish Society of Home Hospitalisation and UK Hospital at Home Society, theses databases (e.g., DART-Europe E-theses Portal, Networked Digital Library of Theses and Dissertations, THES.fr, TESEO) and official healthcare-related institutions (e.g., WHO, European Union Publications Office, Hospital at Home Johns Hopkins University School of Medicine, Agency for Healthcare Research and Quality). The search terms used were: `hospital at home´, `home care´ and `workload´.

### Study selection

Studies that analysed factors associated with the workload of health professionals in HaH were included. The workload could be measured in terms of time or number of visits for each care activity, as well as via tools, models or questionnaires used to predict workload. We took as a reference the HaH definition proposed by the Spanish HaH society [3].

We excluded studies with patients institutionalised in nursing homes or studies that evaluated the workload of primary care physicians or nurses, pharmacists, social workers or informal carers. Articles published in languages other than English, Spanish, German, French, Portuguese, Italian or Greek were not included. Abstracts presented at scientific conferences, letters to the editor and study protocols that did not provide information on factors associated with the burden of care and descriptions of less than 8 cases were also excluded.

### Data extraction and quality assessment

The selection process and quality assessment was carried out by four experienced reviewers, divided into two working groups (KV and MM; NP and EO). Each team independently reviewed the title and abstracts of half of the identified references. Subsequently, those selected studies that met the established selection criteria were reviewed in full text. Any conflicts were solved by consensus and the final decision to include an article was made by agreement between the four reviewers. The following information was extracted for each study: first author, year and location, main objective, study design, setting, study population, workload outcome and significant predictors.

The quality of all retrieved studies was peer-reviewed using the Critical Appraisal Skills Programme (CASP) checklist, which was regarded appropriate considering the design of the included studies. Minors adaptations were made for cross-sectional studies [19]. This tool has been previously used in other systematic reviews [20–24]. Depending on the study design, different variations of the checklist are available including 10 to 12 questions with ‘yes’, ‘no’, ‘cannot tell’ responses and open-ended questions. The items of the checklist are grouped into three sections: A. Are the results of the trial valid?, B. What are the results? and C. Will the results help locally? Each section was assessed as high, moderate, or low quality, corresponding to 2, 1 and 0 points, respectively. The overall study quality was calculated by adding up all three-section scores. Therefore, 5 to 6 points indicated a study of high quality, 3 to 4 points moderate and ≤ 2 low quality [25]. Disagreements regarding the quality scores were discussed and resolved among the same four reviewers.

The certainty of the prognostic factors related to HaH workload was assessed using the GRADEpro tool [26]. The domains of GRADE for rating the certainty of the evidence were: risk of bias, imprecision, inconsistency, indirectness, and publication bias, as well as, large effect, plausible confounding factors and dose response gradient. The certainty assessment was conducted by two experienced reviewers (JAC and NP). Both reviewers conducted the certainty assessment of the main factors related to workload in HaH on the one hand, and the scales used to measure such workload, on the other hand. Disagreements regarding the scores were discussed and resolved between the same two reviewers.

## Results

**Fig. 1:**
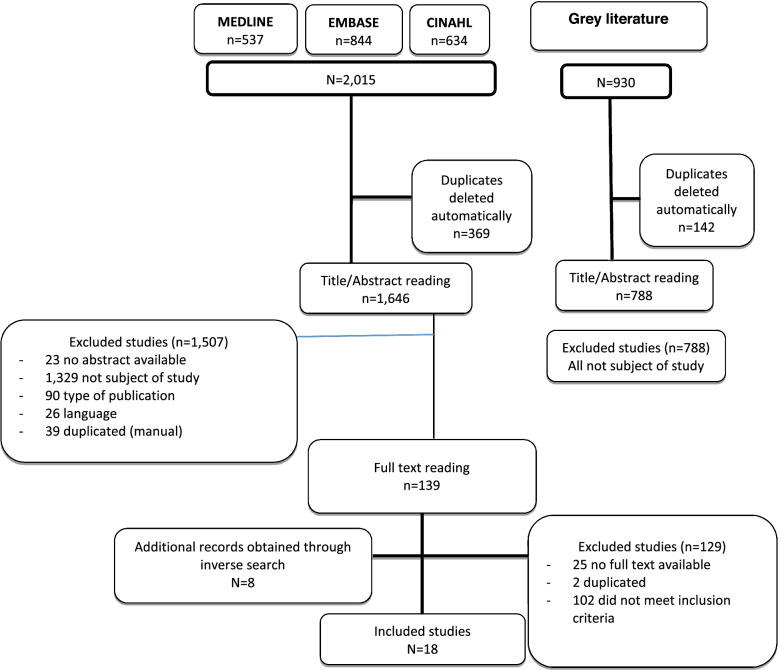
Study flow diagram

For the present systematic review, 2,015 references were retrieved: 537 from MEDLINE, 844 from EMBASE, and 634 from CINAHL. After automatically removing duplicated articles, references were reduced to 1,646. The revision of titles and abstracts resulted in 139 potentially relevant references after removing 1,507 records that did not answer the research question and those that did not fulfil inclusion criteria. After full-text assessment 129 studies were excluded (102 did not meet the selection criteria, two were duplicated and no full text was available for the remaining 25 studies). Eight more articles were identified at this stage by inverse search. No papers meeting the selection criteria were located within the grey literature. Therefore, 18 studies were finally included in the present systematic review (Fig. 1).

### Description of studies

We identified 15 cohort studies of which 11 were prospective [27–37] and four retrospective [38–41]. The remaining three studies were cross-sectional [42–44]. Twelve of them were conducted in the United States [28–31, 33–36, 38, 39, 41, 44], two in Australia [27, 43], and one in each of the following countries: Canada [37], France [32], Netherlands [40], and Norway [42]. Although two studies did not provide data on the characteristics of the study population [33, 42], most of them corresponded to a population over 60 years of age, mostly female. The number of patients included in the selected studies was 56,706 ranging from 50 to 35,232 patients (Table 1).

**Table 1:** Characteristics of included studies (arranged by publication year)

(Reference) First author + Year + Country	Study design	Setting	Study population	Risk of bias	Quality
[38] Harrold, 2014 U.S.A	Retrospective cohort	Hospice Care at Home	35,232 patients (53.8% women). Mean age mean: 79 (71–89) years	Convenience sample	High (5)
[42] Holm SG, 2014 Norway	Cross-sectional	Home Care Services, including Home Help, Home Nursing Care or both	276 home care recipients in municipality A and 181 in B	Convenience sample	High (5)
[27] Montalto M, 2010 Australia	Cohort	HIH, as the equivalent of a ward or clinical unit of the hospital, in a large not-for-profit private Hospital	3,423 episodes (44.7% women); age range 0 to + 80	Convenience sample. There is no control group. There is no control for confounding variables in the design/analysis. There is no blind or third party evaluation. Precision of results not reported (only p values)	Low (2)
[43] Vecchio N, 2007. Australia	Cross-sectional	Community-based non-government national home care services provider	218 clients (57% women), age range 7 to 99	Single-centre study with a convenience sample. Precision of results not reported	Medium (3)
[39] Adams, 2001. U.S.A	Retrospective cohort	Non-profit Medicare-certified Home Health Agencies	2,788 episodes of medical-surgical patients (65% women), mean age (75 years (SD 13.5)	Convenience sample	High (5)
[28] Adams, 2000 U.S.A	Prospective cohort	Non-profit Medicare-certified Home Health Agencies	2,788 episodes of medical-surgical patients (65% women), mean age (75 years (SD 13.5)	Convenience sample. There is no control for confounding variables in the design/analysis	Medium (3)
[29] Lee TT, 2000 U.S.A	Cohort	Home Healthcare	244 patients (50% women), mean age 60.5 years	Single-centre study with a convenience sample. Precision of results not reported	Medium (3)
[30] Payne SM, 1998 U.S.A	Cohort	Non proprietary Home Health Agencies	4,426 home health visits, based on 2,012 clients. The average age of the clients was 59.2 years, 58.5% were women	Convenience sample of experienced nurses (unrepresentative)	Medium (3)
[31] Hays BJ, 1995 U.S.A	Cross-sectional	Home health agency that serves urban and rural areas	237 patients (64% female), 81% 60 years or older	Precision of results not reported	Medium (4)
[32] Bonifassi, 1994. France	Cohort	Hospitalisation at home centers	163 stay in Hospitalisation at home centers. Patients with HIV, with an average age of 36.5 years, mostly male	Convenience sample. Limited to HIV-patients Basic descriptive analysis, without adjusting the results and without reporting its precision	Low (1)
[44] Trisolini MG, 1994. U.S.A	Cross-sectional	Home Healthcare	273 patient visits; 67% women. Mean age: 67.4 (SD: 16.8) years	Convenience sample Non-validated measuring instrument Precision of results not reported	Medium (4)
[40] Tiesinga, 1994. Netherlands	Retrospective cohort	Community Health Care institutions (rural and urban)	65 community health nurses and community nurse auxiliaries; 1,200 patients 69% women); average age: 70 years	Convenience sample Non-validated measuring instrument Precision of results not reported	Medium (4)
[33] Churness, 1991 U.S.A	Cohort	Home Health Nursing Services	83 out of 138 nurses collected data on 1,183 home visits. In phase III, 187 visits were in the sample	Convenience sample During the time of the study, pay-per-visit was introduced (possible information bias). Non-validated measuring instrument Precision of results not reported	Low (2)
[34] Cox CL, 1990 U.S.A	Cohort	A hospital-affiliated, non-profit, Medicare-Certified home health care agency that provides skilled nursing care, and other professional and home health aide services	50 patients (68% women); mean age: 76.8 years	Convenience sample It does not take into account factors related to the activity of professionals. Precision of results not reported	Medium (3)
[35] Williams BC, 1990. U.S.A	Cohort	Home health services	1984 episodes of care corresponding to 1963 patients (63% women); median age: 69 years (range < 1 to > 99 years)	It does not take into account factors related to the activity of professionals. Precision of results not reported	Medium (3)
[36] Peters DA, 1988. U.S.A	Cohort	Home Care, as provided by a visiting nurse association (Agency A) and a hospital-based home care program (Agency B)	560 home care cases: 314 from Agency A and 246 from Agency B Mean age of 63	Convenience sample Validity data of the measuring instrument not shown. Precision of results not reported	Medium (4)
[37] Stark AJ, 1984. Canada	Cohort	Long-term care program	3518 clients (75.9% women); Mean age in Unit A (urban): 78.5 years (S.D. 13.3) and in Unit B (semi-rural): 74.6 years (S.D. 14.8)	Convenience sample There is no definition about the measure of the level of care/activity performed. No analysis plan is established. Precision of results not reported	Medium (3)
[41] Ballard, 1983. U.S.A	Retrospective cohort	Home Care Agencies	397 patient records (56.2% women); mean age: 70.9 years (range 1 to 96)	Limited to cancer or cardiac patients. No information about the professionals’ activities. There is not standardized definitions	Medium (4)

The type of patients studied was diverse and included terminal patients [38], AIDS patients [32], those with various medical or nursing diagnoses [28, 29, 31, 35, 36, 41, 43, 44], unspecified [27, 33, 40, 42], and others [30, 37, 39].

Regarding the characteristics of the service provider organisations, 15 corresponded to Home Care Services [28–31, 33–37, 39, 41–43], two to Hospital at Home [27, 32], and one to Hospice Care [38]. The professionals who participated in the included studies were nurses, health care workers, auxiliaries and others not specified, but none of the studies mentioned physicians. Three of the 18 included studies were classified as high quality [38, 39, 42], 12 as medium [28–31, 34–37, 40, 41, 43, 44], and three as low quality [27, 32, 33] (Table 1). The main risks of bias identified in the included studies were: convenience sampling, lack of control for potential confounding factors, lack of information on the precision of the results, and problems related to external validity.

### Workload and predictive factors

Ten of the 18 included articles measured the workload as time [27, 28, 30, 31, 33, 39, 40, 42–44], and in five articles the workload was measured as the number of visits [35–38, 41]. While three of the included studies presented the workload in both ways  [29, 32, 34] (Table 2).

**Table 2:** Workload and factors related to workload (arranged by publication year)

(Reference) + First author + Year	Workload Outcome	Significant predictors
[38] Harrold J. 2014	Nº visits/day	Increased workload: age < 65, sex male, primary caregiver non spouse, lower Palliative Performance Scale, presence of pain, admitted from a hospital, admitting diagnosis, more than one diagnosis, have a foley catheter, feeding tube, oxygen, pressure ulcer or intravenous access, weekend admission
[42] Holm SG. 2014	Indirect care time/Total care time	Increased workload: more driving time, including transfer time, and more time required to document details of the care given
[27] Montalto M, 2010	Lenght of stay/patient (days/patient)	Higher workload: referred from Hospital wards
[43] Vecchio N. 2007	Care time/patient	Increased workload: ONI: decreasing functional profile and male gender; no nursing services was also associated with increasing allied health time. OPR was found to be less effective as a predictor
[39] Adams CE. 2001	Direct care time (min)/visit	Living in a rural locale increased total direct care time by an average of 150 min after patient characteristics and health status were controlled, in comparison to living in an urban locale
[28] Adams CE. 2000	Direct care time (min/visit	Across the five diagnostic categories, the average RN visit duration of the studies ranged from 48 min in patients with diabetes mellitus and pneumonia to 55 min in orthopedic patients
[29] Lee TT. 2000	Resources utilization (Nº of RN visits, RN hours of care, episode of care and type and number of nursing interventions)/patient	Increased workload: total number of nursing diagnoses and two specific nursing diagnoses (alteration in mobility and knowledge deficit in IV therapy) were strong predictors of overall resource use
[30] Payne SM. 1998	Care time (min)/visit home	Increased workload: admission visit (versus continuing, readmission, or discharge), terminal/care giver factor, and higher Clinical Instability Factor
[31] Hays BJ. 1995	Direct hours of nursing care in the home/patient; mean visit lenght/visit	CHIRS explained a significant (p < 0,001) amount of variation in nursing resource consumption; Omaha PCS significantly predicted direct hours of nursing care
[32] Bonifassi L. 1994	Nº visits/patient/day; Care time/patient/day	Increased workload: lower Karnofsky index, reasons for hospitalization: end of life care
[44] Trisolini MG. 1994	Nursing time/visit	Increased workload: provider-related: new admission; patient-related: zip code, physical therapy/ occupational therapy/ speech therapy support services-receives some of needed; visit-specific: medication problems-prefill, lengthy education, number of telephone calls, expected post-visit telephone calls, expected post-visit paperwork-physician´s orders
[40] Tiesinga L J. 1994	Average visit time per patient	Separates activities explain more variance (39%) of the average visit time per patient than activity categories (29%) or the care types (13%). Of the 87 activities analysed 19 activities were relevant. The activities explain the average visiting time per patient better than the developed care types do
[33] Churness VH. 1991	Direct and indirect nursing care time/visit	The relationship between total score and length of home visits was direct; at best only 46–64% of the variation length among home visits can be accounted for. This instrument can be a useful tool in measuring nurse workload after appropriate adaptations in the specific setting in which it will be used
[34] Cox CL. 1990	Nº visits/patient/episode; Frequency and duration of nurse visits/patient	The only variables that predicted days of service and use of resources were self-care capacity (inability to assume self-care predicted an increase), agency admission diagnoses (neoplasia predicted a decrease), and readmission diagnoses hospital (kidney disease predicted a decrease)
[35] Williams BC. 1990	Nº visits/case/week (Intensity of service)	For intensity of service: ≥ 75 age, and diseases of the blood and blood-forming organs (lower); Diagnosis categories injury and poisoning, diseases of the skin, and the prognosis category good (higher)
[36] Peters DA. 1988	Nº visits/case	CHIRS rating and number of nursing visits were positive correlated (*r* = 0.39, *p* = .000)
[37] Stark AJ. 1984	Nº contacts/client	The final analysis showed that age was the only independent variable: the number of contacts increases with age
[41] Ballard S. 1983	Nursing visits/patient/day	All of the variables were significant and the overall variance accounted for was 19.5%. The Health Status Scale, which measured deficits in daily activities and nursing problems, proved to be the best predictor for agency visits (the higher the score, the greater the use of resources) and contributed to 8% of the variance

We identified a big number of prognostic factors in the included studies (Table S2). The predictive factors related with the workload were diverse and included variables related to patient characteristics (age, gender, functional status, clinical diagnoses, medical device holder like catheter or feeding tube and clinical instability factor), as well as to the characteristics of the institutions where the studies were conducted (rural environment, driving time, visit type, clinical service provided). The following characteristics were associated with higher workloads (Table 2): being older [35, 37–39], being male [38, 39], living in a rural environment [39], presenting a higher number of diagnoses [28, 29], having a worse functional status [32, 38], and being unable to assume self-care [34], among others. One study [42], showed that indirect time (driving time and time required to document details of the care provided) affected the total care time, as it could represent between 31 and 60% of total care time. Tiesinga et al. [40] showed that separate activities explained better the average visit time per patient than grouped activity categories or care types.

Gender and visit type showed moderate certainty of evidence and rural environment high certainty of evidence (Table 3). However, the certainty of evidence was low for age, functional status and clinical diagnoses. The clinical service provided was not considered for certainty evidence assessment because there was a great variation depending on the health system. The clinical instability factor was poorly defined and patient conditions with medical devices are heterogeneous and not comparable to each other. Finally, factors like driving time, transfer time and document time were included in the outcome measure of workload expressed as time attributed to each activity.

**Table 3:** Certainty assessment (Factors)

Nº of studies	Study design	Risk of bias	Inconsistency	Indirectness	Imprecision	Other considerations	Effect	Certainty
Age: [38] Harrold, [39] Adams 2001, [35] Williams, [37] Stark								
4	observational studies	serious^a^	serious^b^	not serious	serious^c^	all plausible residual confounding would reduce the demonstrated effect	Most studies showed increased workload with patient´s age	⨁⨁◯◯ Low
Gender: [38] Harrold, [39] Adams 2001								
2	observational studies	not serious^a^	not serious	not serious	serious^d^	all plausible residual confounding would reduce the demonstrated effect	Higher workload in man	⨁⨁⨁⨁ High
Functional status: [32] Bonifassi, [38] Harrold								
2	observational studies	serious^a^	not serious	Very serious^e,f^	serious^a,c^	all plausible residual confounding would reduce the demonstrated effect dose response gradient	Increased workload in patients with poorer functional status	⨁⨁◯◯ Low
Clinical diagnoses: [38] Harrold J, [28] Adams 2000, [29] Lee, [44] Trisolini, [35] Williams, [34] Cox,								
6	observational studies	serious^a^	not serious	serious^f^	serious^a,c^	all plausible residual confounding would reduce the demonstrated effect	There is an association between workload and the number or type of clinical diagnoses of patients	⨁⨁◯◯ Low
Visit type: [44] Trisolini, [30] Payne								
2	observational studies	serious^a^	Not serious	Serious^f^	Serious^c^	all plausible residual confounding would reduce the demonstrated large effect	New admissions increased workload	⨁⨁⨁◯ Moderate
Rural environment: [39] Adams 2001								
1	observational studies	not serious	not serious	not serious	not serious	strong association all plausible residual confounding would reduce the demonstrated effect dose response gradient	Living in a rural locale increased workload comparison to living in an urban locale	⨁⨁⨁⨁ High

^a^ Convience sample overall the studies

^b^ Different results on effect size, direction of association and significance

^c^ Precision of results not reported in most of the studies

^d^ The standard error of some of the studies is large

^e^ Very specific population. Generalization problems

^f^ Different ways of measuring outcome (workload) and factor

Five studies used validated scales to predict workload: ONI (Ongoing Needs Identification) [43], CHIRS (Community Health Intensity Rating Scale) [31, 36], VNA-LA/USC Home Health Patient Classification System to Home Health [33], and Health Status Scale [41]. CHIRS was useful in the two studies in which it was used (medium quality), explaining a significant part of the variation in the number of nursing care at home hours [31], and significantly correlating with the number of visits per case [36]. Vecchio et al. [43] found that ONI (medium quality) was useful in predicting time of care per patient while OPR was less effective as a predictor. The study by Ballard et al. [41] (medium quality) found that The Health Status Scale, which measures deficits in activities of daily living and nursing problems, was the best predictor of the number of visits per patient and day. All studies using scales as prognostic factors showed low certainty evidence according to GRADE (Table 4).

**Table 4:** Certainty assessment (Scales)

Nº of studies	Study design	Risk of bias	Inconsistency	Indirectness	Imprecision	Other considerations	Effect	Certainty
ONI: [43] Vecchio								
1	observational studies	serious^a,b^	not serious	not serious	serious^c^	none	ONI survey predict nursing and allied health resource requirements for home care services	⨁⨁◯◯ Low
CHIRS: [31] Hays BJ, [36] Peters								
2	observational studies	serious^a,b^	not serious	not serious	serious^c^	none	Workload increased with each increasing level of CHIRS rating CHIRS explains variations on resource consumption	⨁⨁◯◯ Low
Health Status Scale: [41] Ballard								
1	observational studies	serious^d,e^	not serious	serious^f^	serious	all plausible residual confounding would reduce the demonstrated effect	The higher the Health Status Scale score, the greater the use of resources	⨁⨁◯◯ Low
VNA-LA/USC HHPCS: [33] Churness								
1	observational studies	very serious^a,g^	not serious	not serious	serious^c^	all plausible residual confounding would reduce the demonstrated effect	The is a relationship between total score and workload	⨁⨁◯◯ Low

^a^ Convience sample

^b^ Single centre study

^c^ Precision of results not reported

^d^ Validity data of the measuring instrument not shown

^e^ Lack of sample size estimation

^f^ Very specific population. Generalization problems

^g^ Posible information bias

## Discussion

The reviewed literature shows that, according the used definition of HaH, highly complex patients with time-intensive needs require a significant contribution of health professionals with higher intensity [45, 46]. However, in addition to these patient-oriented measures, the amount of work required for each case should also be considered. Both, the workload and its predictive factors were evaluated in different ways across the included studies. This is probably due to the type of patients, organisations and health professionals studied. In the present systematic review, workload was mainly studied in nurses but not in physicians. The reviewed studies were focussed in home health services and included a very diverse range of patients.

According to the results derived from the included studies, male sex, greater age, worse functional status, greater number of diagnoses and living a rural environment are some of the independent predictors of workload.

Similar results were also found in other studies. Thus, according to a longitudinal study carried out in 1,068 patients over 64 years from a home care program [47], risk factors for receiving more nursing visits at home were male gender, dependency for daily activities decubitus ulcers and receiving emergency medical care at home. In contrast, patients with major cognitive impairment had a lower probability of receiving nursing visits at home. Marek et al. [48] showed that age < 65 years was significantly correlated with the hours of nursing care. Medical conditions such as, mental disorders, musculoskeletal diseases and endocrine disorders, as well as sociodemographic factors, were related to visits, hours or days in home health care [48, 49].

The patient classification systems used to predict workload were valid and reliable, but they had some limitations for their generalisation to different settings. Tools such as CHIRS [31, 36] (medium quality) or ONI [43] (medium quality) showed agency or region-specific differences that might limit the generalisation and the applicability of the data.

Therefore, several factors related to disease severity and comorbidity have been identified as being associated with workload, although these results are very much influenced by the organisation system used and by the assessment of the patients’ needs carried out [48–50].

In the present systematic review, the studies by Trisolini [44] and Ballard [41] showed that the characteristics of the agencies were a significant predictive factor of the workload, although without clearly identifying those specific characteristics and what role they play. The authors suggest that the effect of the agencies´ characteristics could be due to administrative procedures, the training and practice style of the health professionals, the method of payment (per visit or per hour) and other organisational factors that should be explored and analysed in greater detail. The results of the study by Marek et al. [48] indicated that private insurance, as primary payment source, was a significant predictor of home health care visits, accounting for 3% of the variance.

Among the 18 included studies only two strictly corresponded to HaH definition used by Shepperd et al. [7]. This was because with the heterogeneity of HaH models and the terms we found in the available scientific evidence, we established a broad definition of HaH based on the definition by Spanish HaH society. Nevertheless, despite the great diversity of care models, some common characteristics are shared by all most of them. Therefore, the identified factors related to workload of professionals would be useful and applicable to many care models although not to all.

We also found different ways of measuring `workload´, which complicated the assessment of the comparability of the studies and decisions on staffing needs and costs [27, 44]. First of all, the type of activity performed by health care workers (doctors and nurses) in a HaH unit may be different depending on the composition of the care teams, which undoubtedly impacted on the associated workload. We did not found studies that measured the workload of medical doctors in HaH. Secondly, it should be borne in mind that there are different ways to measure workload: through the numbers of visits or through time spent with each patient. A limitation of using the number of visits is that it is assumed that each visit lasts the same amount of time and thus, does not account for the variation in visit time. In addition, time can be measured as direct or indirect time. Moreover, indirect time has a great impact on the total care time as it can represent between 31 and 60% of total care time [42]. We believe that, the frequency of visits and their duration should be measured in order to determine the workload, as well as the direct and indirect time of the health professionals that perform this type of care. However, it is understandable that measuring time is hardly feasible in practice.

On the other hand, work intensity and workload [51, 52] are closely related concepts that are often used interchangeably although they are not equivalent, leading to confusion: the former is a concept closely related to `patient dependence´, `acuity´ and `severity´, while the latter also includes indirect time, factors unrelated to patients such as, contextual and/or organisational factors and staff-related factors [53].

It should be pointed out that due to the diversity of predictors and ways of measuring workload it has not been possible to summarise the results of the included studies numerically. Additionally, a meta-analysis was not conducted since data synthesis was not indicated. Nevertheless, the significant predictive factors for workload at HaH identified in the included studies were summarised in Table 2.

Among the strengths of our systematic review, we could point out that the work was conducted following the standardised PRISMA methodology. It is also important to highlight that studies written in a range or languages other than English were included and the time period established for the search was very broad.

Lastly, most of the 18 included studies used an appropriate design for the assessment of workload predictors (cohort). Twelve of them were moderate quality and the three high quality studies were carried out in recent years. The quality assessment of the included studies was carried out through the application of tools (CASP) that allowed the discussion and consensus between researchers. Slight adaptations were made for the assessment of cross-sectional studies. The CASP tool has been increasingly used in last years for the quality assessment of articles for systematic reviews and meta-analyses, due to its versatility and completeness [19–24].

Three of the six prognosis factors assessed showed high or moderate certainty evidence, nevertheless all the scales studied as factors showed low evidence.

## Conclusions

1. The literature on the factors associated with the workload in HaH is scarce for nursing care and null for medical care.
2. The factors associated with higher workloads were: being older, male, living in a rural environment, presenting a higher number of diagnoses, having worse functional status and being unable to assume self-care.
3. There is moderate or high evidence of increased workload in patients living in a rural environment, males, and newly admitted. All other studied factors showed a low certainty of evidence on their association with workload.
4. The identified predictors of workload are mostly associated with home nursing care modalities, and, although many of these factors could affect the workloads of HaH professionals, whichever the model of HaH we refer to or the HaH definition used, it may not be applicable to all models types.
5. In order to measure the workload in HaH, the frequency of visits and their duration should be measured and both, direct and indirect time that health professionals spend performing this type of home care.
6. More studies that include physicians and proxy measures of workload are needed.

## Supplementary Information

**Additional file 1:** **Table S1.** Search strategy.

**Additional file 2.** **Table S2.** Objective of included studies and predictive factors (arranged by publication year).

## Data Availability

The datasets used and/or analysed during the current study are available from the corresponding author on reasonable request.
