# Supplementary material for: Factors associated with the workload of health professionals in hospital at home: a systematic review
Source: BMC Health Serv Res. 2022 May 26;22:704. doi: 10.1186/s12913-022-08100-4 (PMC9134652; doi:10.1186/s12913-022-08100-4)
Supplement: Supplementary file 1 — Additional file 1: Table S1. Search strategy. [file 12913_2022_8100_MOESM1_ESM.pdf]

Table S1. Search strategy

Database: Ovid MEDLINE(R) and EMBASE

----

1 exp Home Care Services/
2 exp Home Care Services, Hospital-Based/
3 *Home Health Nursing/
4 hospital at home.mp.
5 home hospitalization.mp.
6 hospital home care.mp.
7 hospital in the home.mp.
8 hospital in home.mp.
9 in-home program.mp.
10 in-home hospitalization.mp.
11 in-home healthcare service*.mp.
12 home medical care.mp.
13 home care surveillance.mp.
14 home-care surveillance.mp.
15 home care hospitalization.mp.
16 home-care hospitalization.mp.
17 home-care program*.mp.
18 home care program*.mp.
19 hospital-based home care.mp.
20 hospital based home care.mp.
21 hospital based home nursing care.mp.
22 hospital-based home nursing care.mp.
23 hospital based home nursing.mp.
24 hospital-based home nursing.mp.
25 home healthcare.mp.
26 home health care.mp.
27 home visit*.mp.
28 home health care nursing.mp.
29 home healthcare nursing.mp.
30 home nursing care.mp.
31 home health nursing.mp.
32 domiciliary care.mp.
33 domiciliary healthcare.mp.
34 domiciliary health care.mp.
35 domiciliary hospitalization.mp.
36 1 or 2 or 3 or 4 or 5 or 6 or 7 or 8 or 9 or 10 or 11 or 12 or 13 or 14 or 15 or 16 or 17 or 18 or 19 or 20 or 21 or 22 or 23 or 24 or 25 or 26 or 27 or 28 or 29 or 30 or 31 or 32 or 33 or 34 or 35

37 *Workload/
38 *Health Workforce/
39 *Time Management/
40 exp "Task Performance and Analysis"/
41 workload*.mp.
42 work load*.mp.
43 nursing workload*.mp.
44 nursing work load*.mp.
45 care hours.mp.
46 nursing time.mp.
47 nursing care time.mp.
48 nursing intensity.mp.
49 nursing care intensity.mp.
50 nursing work intensity.mp.
51 nursing activities.mp.
52 clinician workload*.mp.
53 clinician work load*.mp.
54 clinician work intensity.mp.
55 clinician care intensity.mp.
56 clinician time.mp.
57 clinician care time.mp.
58 medical workload*.mp.
59 medical work load*.mp.
60 medical work intensity.mp.
61 medical time.mp.
62 medical care time.mp.
63 medical care intensity.mp.
64 doctor workload*.mp.
65 doctor work load*.mp.
66 doctor work intensity.mp.
67 doctor time.mp.
68 doctor care time.mp.
69 doctor care intensity.mp.
70 physician workload*.mp.
71 physician work load*.mp.
72 physician time.mp.
73 physician care time.mp.
74 physician care intensity.mp.
75 physician work intensity.mp.
76 working conditions.mp.

77 working hours.mp.

78 *Work Capacity Evaluation/

79 exp Occupational Stress/

80 work pressure.mp.

81 37 or 38 or 39 or 40 or 41 or 42 or 43 or 44 or 45 or 46 or 47 or 48 or 49 or 50 or 51 or 52 or 53 or 54 or 55 or 56 or 57 or 58 or 59 or 60 or 61 or 62 or 63 or 64 or 65 or 66 or 67 or 68 or 69 or 70 or 71 or 72 or 73 or 74 or 75 or 76 or 77 or 78 or 79 or 80

82 36 and 81

83 exp "Weights and Measures"/

84 exp "Surveys and Questionnaires"/

85 measure*.mp.

86 scale*.mp.

87 score*.mp.

88 survey*.mp.

89 questionnaire*.mp.

90 tool*.mp.

91 83 or 84 or 85 or 86 or 87 or 88 or 89 or 90

92 82 and 91

Database: CINAHL

S11 S8 AND S9 AND S10 -

S10 SU (weights and measures) OR SU (survey or questionnaire) OR TX measure* OR TX scale* OR TX score* OR TX survey* OR TX questionnaire* OR TX tool*

S9 S4 OR S5 OR S6 OR S7

S8 S1 OR S2 OR S3

S7 TX physician care time OR TX physician care intensity OR TX physician work intensity OR TX working conditions OR TX working hours OR SU work capacity evaluation OR SU occupational stress OR TX work pressure

S6 TX medical time OR TX medical care time OR TX medical care intensity OR TX doctor workload OR TX doctor work load OR TX doctor work intensity OR TX doctor time OR TX doctor care time OR TX doctor care intensity OR TX physician workload OR TX physician work load OR TX physician time

S5 TX nursing care intensity OR TX nursing work intensity OR TX nursing activities OR TX clinician workload OR TX clinician work load OR TX clinician work intensity OR TX clinician care intensity OR TX clinician time OR TX clinician care time OR TX medical workload OR TX medical work load OR TX medical work intensity

S4 SU workload OR SU health workforce OR SU time management OR SU (task performance and analysis) OR TX workload OR TX work load OR TX nursing

workload OR TX nursing work load OR TX care hours OR TX nursing times OR TX nursing care time OR TX nursing intensity

S3 TX home healthcare OR TX home health care OR TX home visit* OR TX home health care nursing OR TX home healthcare nurse OR TX home nursing care OR TX home health nursing OR TX domiciliary care OR TX domiciliary healthcare OR TX domiciliary health care OR TX domiciliary hospitalization

S2 TX home care surveillance OR TX home-care surveillance OR TX home care hospitalization OR TX home-care hospitalization OR TX home-care program OR TX home care program OR TX hospital-based home care OR TX hospital based home care OR TX hospital based home nursing care OR TX hospital-based home nursing care OR TX hospital based home nursing OR TX hospital-based home nursing

S1 SU home care services OR SU home care services, hospital-based OR SU home health nursing OR TX hospital at home OR TX home hospitalization OR TX hospital home care OR TX hospital in the home OR TX hospital in home OR TX in-home program OR TX in-home hospitalization OR TX in home health care OR TX home medical care
