# Supplementary material for: Factors associated with the workload of health professionals in hospital at home: a systematic review
Source: BMC Health Serv Res. 2022 May 26;22:704. doi: 10.1186/s12913-022-08100-4 (PMC9134652; doi:10.1186/s12913-022-08100-4)
Supplement: Supplementary file 2 — Additional file 2. Table S2. Objective of included studies and predictive factors (arranged by publication year). [file 12913_2022_8100_MOESM2_ESM.pdf]

Table S2. Objective of included studies and predictive factors (arranged by publication year).

First author + Year + (Reference)	Main objective	Predictive factors considered in the study
Harrold, 2014. (38)	To identify patient characteristics that are associated with the frequency of health care provider visits in the first 8 days of hospice care.	Age, gender, race, admitting diagnosis, admitted from hospital, more than one diagnosis, Palliative Performance Scale (PPS), spouse caregiver, foley catheter, oxygen, weekend admission, advanced directive, pressure ulcer, pain, lenght of stay, intravenous access, feeding tube, pressure ulcer, intravenous access.
Holm SG, 2014. (42)	To learn how nurses and health workers in home-care services spent their working time using GIS to analyze a weekly plan of daily schedules and driving routes, and in particular to calculate distance driven and the amount of time spent driving,	Driving time, including transfer time, and time required to document details of the care given are underestimated in weekly planning schedules and therefore taking them into account increases the total time of actual care
Montalto M, 2010. (27)	To determine whether: 1) Hospital in the Home (HIH) is a safe and effective method of delivering acute hospital care, under usual operating conditions in an established unit; and 2) what patient, condition and treatment variables contribute to a greater risk of failure.	Referred from (Emergency Departments/Direct from rooms/Hospital wards)
Vecchio N, 2007. (43)	To assess the use of the ONI (Ongoing Needs Identification) survey to predict resource requirements in the home care sector.	ONI (Ongoing Needs Identification survey) indicators and OPR (ONI Priority Rating score). Sex, respiratory system, Musculoskeletal/connective tissue, Renal/urinary conditions, Allied health services, Functional profile, Personal care.
Adams, 2001. (39)	To determine if health status differed between rural versus urban home health patients and to identify if locale was a significant predictor of home health direct care time.	Locale (urban vs rural); Covariates: patient characteristics and health status.
Adams, 2000. (28)	To publish information on direct care time by discipline for home health patients as a total group and common diagnoses seen in home health patients.	Five diagnostic categories: Cardiovascular, Orthopedic, Chronic obstructive lung disease, Diabetes mellitus and Pneumonia.
Lee TT, 2000, (29)	To identify patient profile variables that explains variation in resource utilization and outcomes for home healthcare.	Patient profile variables (age, sex, race, marital status, living status, payment source, nursing diagnoses, medical diagnoses, and prognosis).
Payne SM, 1998. (30)	1) To compare visit length across four categories of skilled nursing home health visits and 2) To identify factors influencing visit length.	Independent variable: categories of skilled nursing home health visits (AIDS-related, Hospice/Terminal (HT), IntraVenous (IV) therapy, and Maternal and Child Health (MCH); covariates: 74 variables formed 23 factors.
Hays BJ, 1995. (31)	To explore the relationship between nursing care requirements, using both an intensity index (CHIRS) and nursing diagnoses (Omaha PCS), and nursing resource consumption in home health care.	Community Health Intensity Rating Scale (CHIRS); Omaha Problem Classification Scheme (PCS)

First author + Year + (Reference)	Main objective	Predictive factors considered in the study
Bonifassi, 1994. (32)	To present, among the results of this study, those related to nursing, in addition to the work already published for traditional and day hospitalisation.	Karnofsky index, reasons for hospitalisation
Trisolini MG, 1994. (44)	To identify the factors most useful for classifying visits in terms of visit length.	5 categories, 55 variables: Provider-related (6 variables), Patient´s socioeconomic status (6 variables), Patient´s clinical status (16 variables), Patient´s support services (7 variables), Visit-specific (20 variables).
Tiesinga, 1994. (40)	To explore if a factor evaluation analysis based on the performed activities explains the average visit time per patient better than a previous developed prototype system.	Activities performed during the home visit. 8 categories, 87 activities: Hygienic care, household activities, technical nursing care, health education, support the patient, support caregivers, observation activities, administration activities.
Churness, 1991. (33)	Design, development and validation of the VNA-LA/USC Home Health Patient Classification System for home health.	VNA-LA/USC Home Health Patient Classification System to Home Health.
Cox CL, 1990. (34)	To explore the utility of using patient record data in predicting agency resource use and patient outcome.	Primary diagnosis, secondary diagnosis, agency admission prognosis, hospital readmission diagnoses, primary care giver in home
Williams BC, 1990. (35)	To examine the feasibility of using routinely collected information on patients enrolled in home health care to predict their subsequent use of services.	Age, sex, Medicare and Medicaid enrollement, referral source, medical dagnosis and prognosis
Peters DA, 1988. (36)	To develop and validate a patient classification method for community health that would enable the categorization of patients based on their nursing care requirements.	Community Health Intensity Rating Scale (CHIRS)
Stark AJ, 1984. (37)	To identify variables associated with high workload clients because these persons generate higher than average manpower costs.	Health unit of residence (Urban/semi rural), Client status (Original/New), Age (0-64, 65-74, 75-84, 85+), Level of care at admission (Personal Care/Intermediate care/Extended care), Memory (good/fair/poor/very poor) at admission, Emotional stability (good/fair/poor/very poor) at admission, Hospitalisation (yes/no).
Ballard, 1983. (41)	To determine what factors were most predictive of the quantity of nursing service and total agency service required by cardiac and cancer patients in home-care agencies.	Agency providing services, age, sex, payment source, marital status, primary-care provider, support system, discharge status, surgery, number of diagnoses, use of other professional and nonprofessional services and patient total score on the Health Status Scale (18 items; 1-no problem/2-minor problem/3-oderate problems (outside assistance required)/4-serious problems (maximum assistance required).
